# Brain nutrient sensing: A unifying framework

**DOI:** 10.1146/annurev-physiol-042324-100329

**Published:** 2025-11-10

**Authors:** Clemence Blouet, Gary J Schwartz

**Affiliations:** 1 Institute of Metabolic Science Metabolic Research Laboratories, University of Cambridge, Cambridge CB2 0QQ, UK; 2 MRC Metabolic Diseases Unit, Institute of Metabolic Science Metabolic Research Laboratories, University of Cambridge, Cambridge CB2 0QQ, UK; 3 Departments of Medicine, Neuroscience, and Psychiatry & Behavioral Sciences, Albert Einstein College of Medicine; 4 Fleischer Institute of Diabetes and Metabolism

**Keywords:** nutrient sensing, homeostasis, hypothalamus, obesity, type 2 diabetes, brain

## Abstract

Whole-body nutrient homeostasis is critical for healthy growth, successful reproduction, and survival. We propose a conceptual framework emphasizing the role of brain nutrient-sensing in mediating adaptive responses for the maintenance of nutrient homeostasis. Specialized brain nutrient-sensing cells monitor nutrients and meal-related signals and provide feedback responses to maintain internal nutrient availability and adapt physiological functions according to environmental nutrient fluctuations. Maladaptive functioning of these pathways, may underlie multiple pathophysiological conditions, including cardiometabolic and neurodegenerative diseases. By examining recent advances, this review highlights the importance of brain nutrient sensing in adaptive systemic physiology and behavior, and explores the potential of these neural pathways as therapeutic targets extending beyond obesity management. Ultimately, the goal of this review is to synthesize current evidence into a coherent framework that guides new mechanistic hypotheses, facilitating deeper investigation into how brain nutrient sensing influences health and contributes to disease pathogenesis.

## Introduction

The concept of brain nutrient sensing emerged with Mayer’s glucostatic theory of feeding and the demonstration that neuronal electrical activity in the hypothalamus rapidly responds to changes in blood glucose concentrations (1; 2). These seminal studies provided the first lines of evidence that specialised brain cells can sense changes in glycaemia and respond by recruiting downstream neural circuits. Although the initial hypothesis that brain glucose sensing serves the control of food intake remains debated (3), modern investigations of brain nutrient sensing are still pursued within a conceptual framework directly inspired by Mayer’s intuition that brain glucose sensing engages feedback circuitry that ultimately acts to restore whole-body glucose homeostasis.

A range of nutrient sensing mechanisms have since been implicated in brain sensing of not only glucose, the brain’s main energy substrate, but also fatty acids, amino acids and their metabolites. These mechanisms engage central circuits that act to modulate nutrient intake, peripheral metabolism and inter-organ nutrient fluxes. In this review, we aim to explore the evidence supporting the hypothesis that brain nutrient sensing mechanisms play a major role in maintaining organismal nutrient homeostasis by modulating behavioral and physiological determinants of energy balance as a function of environmental nutrient availability.

## 1 What are the Biological Bases of Nutrient Sensing?

Specialized nutrient-sensing cells in the brain use extracellular nutrients not just as fuels, but also as signalling molecules reporting whole-body nutrient availability. They adapt their functional output to mediate both central and peripheral responses to changes in nutrient availability through behavioural, autonomic, neuroendocrine, somatic and cognitive effectors. These adaptive responses maintain whole body nutrient homeostasis in the face of changes in environmental nutrient availability.

The functional output of brain nutrient sensors depends on ambient levels of nutrients and meal-induced hormones, as well as neurophysiological inputs from peripheral and central sources that come into direct contact with nutrients during the course of a meal. These include the gut and the hepatic portal vein, whose inputs to the brain array of nutrient sensing circuits occur via both the afferent vagus and non-vagal, splanchnic afferents. Collectively the response properties of these gut and portal afferents demonstrate considerable heterogeneity across nutrient chemical sensitivity and molecular phenotypes. Furthermore, they project a distributed representation of nutrient-based information to the CNS along the gut-brain axis, as outlined below.

Importantly, brain nutrient sensing does not include brain-ubiquitous energy sensing mechanisms recruited to match fuel availability to circuit activity, an energy consuming process. For example, visual circuits adapt AMPA receptor conductance to energy levels, reducing synaptic ATP use in conditions of energy deficit through increased activity of the energy sensing AMP-activated protein kinase (AMPK) signalling pathway (4). In this context, energy sensing allows an efficient management of the brain’s energy economy, reducing costly neural functions during food scarcity, but does not contribute per se part to brain nutrient sensing mechanisms as defined here.

**Figure 1:**
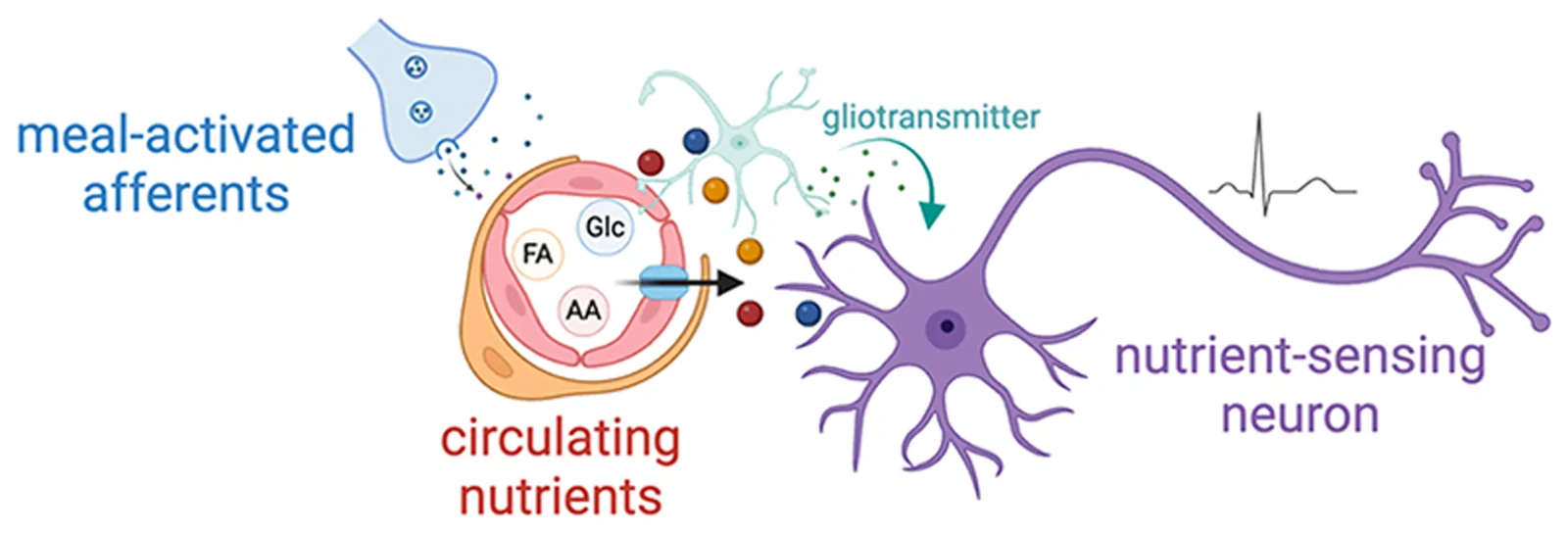
The nutrient sensing niche. Brain nutrient sensing occurs within a unifying nutrient-sensing niche which includes: 1) vascular and barrier cells of the blood-brain barrier (BBB) and in circumventricular organs (CVOs), which modulate the rate and range of nutrient access to the niche, 2) non-neuronal cells, which support circuit activity, mediate structural remodelling within the niche and have direct nutrient-sensing capacity, 3) nutrient-sensing afferents, and 4) nutrient-sensing neurons, which produce the electrical, neurotransmitter and neuropeptide signals modulating the activity of downstream effector circuits. FA: fatty acids, Glc: glucose, AA: amino acids.

Finally, the nutrient-sensing niche should be considered as *the* functionally important unit of investigation, including the neurovascular and glial environment surrounding nutrient-sensing neurons (Figure 1). Structural remodelling of nutrient-sensing niches in response to changes in nutrient availability are an essential aspect to the effects of metabolic states on nutrient sensing (5).

### 1.1 Brain nutrient sensing in neurons

Brain nutrient sensing has been primarily described to occur through neurons able to change their electrical activity, neurotransmitter and neuropeptide release in response to physiological changes in systemic nutrient levels (6). In addition to sensing nutrient concentrations per se, nutrient sensing neurons also track the rate of change in blood nutrient levels. For example, the neuronal activity of glucose sensing neurons in the ventromedial hypothalamus (VMH) or in the lateral hypothalamus (LH) does not correlate with blood glucose levels per se, but instead with the arteriovenous change in glucose levels (7). Detection of different rates of glucose concentration changes can determine distinct functional consequences. This has been observed in the rat caudal brainstem, where slow but not rapid rates of hypoglycemia activate ventrolateral medullary catecholaminergic projections to drive hypothalamic sympathoadrenal counterregulatory responses (8).

Nutrient sensing neurons can adapt their electrophysiological properties in response to changes in nutrient availability. For example, the frequency and amplitude of glucose-mediated spontaneous excitatory postsynaptic currents can vary as a function of the phase of glucoprivation (9). Likewise, physiological changes in glucose concentrations bidirectionally alter the electrical excitability of melanin concentrating hormone (MCH) and orexin glucose-sensing neurons by increasing their membrane resistance (6). In addition, nutrient-sensing neurons experience significant synaptic remodelling and spinogenesis in response to changes in nutrient availability (10; 11).

### 1.2 Brain nutrient-sensing in non-neuronal cells

Non-neuronal cells are increasingly proposed to play an active role in brain nutrient sensing and the regulation of energy homeostasis, but a major challenge lies in demonstrating that the function of non-neuronal cells extends beyond their ubiquitous roles as support cells for neurons. Accordingly, with this caveat in mind, we will examine nutrient-sensing in non-neuronal cells in more detail.

#### 1.2.1 Non neuronal cells mediate brain access to circulating nutrients

Non-neuronal cells form the interface between the blood and the brain parenchyma, both at the level of the blood-brain barrier (BBB) and in circumventricular organs (CVOs), including the median eminence (ME) and area postrema (AP). They regulate the access of circulating molecules to the brain through diverse mechanisms as outlined below.

Brain blood flow, a biophysical regulator of brain nutrient access, is dynamically regulated by glial nutrient sensing. For instance, leptin and low-glucose sensing in pericytes increase cerebral blood flow, resulting in enhanced access of blood-borne products to the brain (12; 13). Likewise, insulin signalling in astrocytes regulates brain blood flow and brain access to insulin and glucose (14). Importantly, reductions in hypothalamic blood flow velocity decrease the orexigenic action of ghrelin by delaying ghrelin’s access to its target neurons (15), highlighting the functional relevance of this regulation in brain nutrient sensing. However, further studies are needed to clarify whether these changes occur under physiological conditions and are selectively targeted to brain regions enriched in nutrient-sensing neurons.

Glial nutrient sensing also induces structural adaptations in the BBB and CVOs, which modulate barrier diffusion properties. For example, increased vascular endothelial growth factor (VEGF) signaling in CVO endothelial cells during fasting or hypoglycaemia enhances capillary fenestration and diffusion of meal-related signals such as ghrelin or glucagon-like peptide 1 (GLP1) into the ventral hypothalamus (16; 17). Nutrient-induced remodelling of the extracellular matrix (ECM) in the median eminence (ME, CVO located at the base of the mediobasal hypothalamus) regulates extracellular diffusion of circulating molecules from the blood to the hypothalamus (18; 19). Production of new oligodendrocytes is important for this plasticity (19). Hypoglycaemia rapidly upregulates oligodendrocyte differentiation in the ME, leading to local ECM remodelling, increased neuronal glucose sensing, and activation of neuroendocrine responses (20). Thus, non-neuronal cells mediate important structural adaptations within nutrient sensing niches, important for the ability of nutrient-sensing neurons to respond to fluctuations in circulating nutrient levels.

ME tanycytes have been proposed to provide an additional contribution through the transport of metabolic hormones from the blood to the brain via transcytosis. For example, the hypothalamic access, neuronal activation and weight loss actions of GLP1 mimetics are reduced in mice with inducible tanycyte-specific inhibition of SNARE-mediated exocytosis (21). However, whether tanycytes express the transporters required to mediate transcytosis remains controversial (22).

#### 1.2.2 Nutrient metabolism in non-neuronal cells, neuroglial metabolic coupling, and brain nutrient sensing

Non-neuronal cells maintain trophic support to neurons, which they achieve through activity-dependent neuroglial metabolite shuttles, such as the glucose-lactate shuttle (23), required for neuronal excitability, synaptic transmission (24), and neuronal survival (25). Perturbations in glial intracellular nutrient metabolism and release of lactate or other metabolites inevitably produce significant consequences on neuronal activity and the execution of diverse brain functions.

Importantly for the present argument, several studies have reported impaired *systemic* energy and glucose homeostasis following genetic deletion of nutrient transporters or metabolic enzymes in astrocytes or tanycytes. For example, inhibition of astrocytic glucose sensing impairs glial lactate release, hypothalamic glucose sensing and systemic energy and glucose homeostasis (26; 27). These responses could reflect the role of glia in supporting the energetic needs of local circuits, crucial during hypoglycemia or high energy demand (28; 29). Likewise, glial adenosine release modulates energy intake, for example through the inhibition of agouti-related peptide (AGRP) neurons (30). However, the inhibitory neuromodulation of adenosine reduces global brain metabolic activity (31), and thus might not represent a specific nutrient sensing mechanism from the standpoint of distal or downstream effector functions. Strikingly, optogenetic stimulation of tanycytes activates both AGRP and proopiomelanocortin (POMC) neurons, which have opposite effects on appetite, suggesting non-specific neuronal activation independent of the downstream functional consequences (32). From this body of work, it remains a challenge to determine the degree to which non-neuronal cells contribute to brain nutrient sensing through mechanisms beyond their ubiquitous role in metabolic support to neurons or energy-dependent neuromodulation.

Neuroglial metabolic coupling through fatty acid metabolites also plays an important role in neuronal functions. Brain lipid homeostasis and synaptic plasticity rely on astrocytic lipid metabolism (33; 34), and astrocyte lipid storage in lipid droplets protects neurons from lipotoxicity and oxidative stress under normal conditions (35). In the hypothalamus, astrocyte lipid uptake, storage, and metabolism have also been proposed to regulate energy homeostasis, but whether this is independent of neuroglial lipid coupling is unclear (36).

#### 1.2.3 Glial signalling molecules and brain nutrient sensing

An emerging literature suggests that glial nutrient sensing can generate specific signalling molecules encoding changes in peripheral nutrient availability and informing local circuits of these changes. For example, fasting induces tanycytic expression and release of FGF21, and deletion of tanycytic *Fgf21* disrupts peripheral lipid and glucose homeostasis (37). The exact neuronal mechanisms engaged to produce these peripheral responses are unclear, but FGF21 mediated increases in the expression of hypothalamic arginine-vasopressin and thyroid-stimulating hormone suggests a local regulation of neuroendocrine output.

The production and release of glial endozepines such as octadecaneuropeptide (ODN) increase with peripheral glucose or insulin administration (38). Acutely, ODN signaling activates melanocortinergic feeding circuits, leading to a decrease in food intake (39). Bidirectional changes in astrocytic ODN synthesis also modulate weight gain in high-fat fed mice, suggesting that this pathway is important in long-term energy homeostasis. Of note, ODN signaling has minimal effect in chow-fed mice, and seems specifically recruited during periods of high energy demand, such as during cold exposure or after a period of energy deficit (40).

Astrocytic release of bioactive lipids is also emerging as an important contributor to brain nutrient sensing. Specifically, astrocytes release prostaglandin E2 (PGE2) in response to nutritional signals such as ghrelin and leptin, promoting synaptic remodelling in AGRP neurons (41). Hyperglycemia has also been associated with increased PGE2 release in the hypothalamus, where it modulates the activity of VMH glucoregulatory circuits (42). Furthermore, the excitatory effects of PGE2 have been observed across various neuronal populations such as hypothalamus GnRH neurons (43).

### 1.3 Neuroanatomical distribution of nutrient sensing

#### 1.3.1 Peripheral nutrient-sensing inputs to the brain

For the purposes of this review, nutrient sensors from the oral cavity, gut and hepatic portal vein whose neurophysiological activity is acutely modulated by nutrients during a meal are considered to provide a primary neural source of peripheral meal-related nutrient information to the brain. Both vagal and spinal sensory nerves supply key upper gastrointestinal and hepatic sites that are exposed to and activated by the mechanical and chemical properties of ingested food, the digestive products of a meal, and prandial gut hormonal/neuropeptide/neurotransmitter secretion.

Importantly for the central representation of nutrient availability at the level of the cmNTS, individual gut-innervating fibers can respond to individual meal-related stimuli. Single vagal afferent fibers supplying the stomach or duodenum are excited, respectively, by gastric loads or duodenal nutrient infusions at volumes and rates that mimic the rates of oral intake and gastric emptying of consumed nutrients (44), (45). This segregation of gastric load and small intestinal nutrient signals appears to be maintained across projections to distinct neuronal populations of the caudomedial nucleus of the solitari tract (cmNTS) (46; 47). GLP1 receptor (GLP1R) expressing gut vagal afferents encoding both gastric and duodenal stretch project to dorsal and mediolateral terminal fields of the cmNTS, while GPR65 vagal afferent neurons responsive to small intestinal nutrient infusions project to a neuroanatomically distinct ventromedial region of the cmNTS (46). These two segregated terminal field populations have been implicated in distinct feeding and glucoregulatory functions across two neurochemically distinct cmNTS subpopulations (48); stimulation of GLP1R expressing fibers primarily activate cholecystokinin (CCK) cmNTS neurons and potently suppresses feeding, while stimulation of GPR65 afferents primarily activate dopamine beta-hydroxylase (DBH) cmNTS neurons and modulates systemic glucose availability by reducing hepatic glucose production (48).

These findings align with the more general notion that increases in nutrient availability, through feeding or gastrointestinal nutrient infusions, drives neurochemically distinct populations of cmNTS neurons via vagal afferents to regulate behavioral and physiological determinants of nutrient uptake (e.g, (49)). For example, vagally driven prolactin releasing hormone (PRLH) neurons of the cmNTS are rapidly activated by the taste of food on a moment-to-moment basis to limit the rate of ingestion, and this activation can be dissociated from the post-oral gastrointestinal consequences of nutrient availability (50). In contrast, preproglucagon (PPG) cmNTS neuronal activity tracks cumulative food intake during a meal, and activation of these neurons limits meal size (50). Taken together, these studies suggest there are distinct neural circuitries downstream from these nutrient responsive neurons that mediate distinct behavioral outcomes.

#### 1.3.2 Central nutrient-sensing loci

Neurons with nutrient-sensing properties, i.e. those that are able to alter their neurophysiological activity characteristics in response to changes in extracellular nutrient levels, have been reported in a number of areas in the brain, including not only hypothalamic (ARH, VMH, LH, paraventricular hypothalamus-PVH) and hindbrain neurons (area postrema-AP, cmNTS) but also forebrain limbic, striatal and cortical regions (nucleus accumbens, ventral tegmental area - VTA), amygdala , hippocampus, prefrontal cortex), suggesting that nutrient availability can modulate the activity of a wide neuroanatomical and neurochemical range of brain circuits (51). Whether and how these nutrient sensing populations are engaged in healthy physiology remains controversial, in part because the supraphysiological concentrations of nutrient stimuli have historically been applied to identify nutrient responsiveness. In this regard, it is critical to recall that for most nutrient-sensing neurons, BBB transport mechanisms regulate access to circulating nutrients. These mechanisms tend to delay and dampen acute meal-induced changes in circulating nutrient concentrations (52). However, some brain nutrient sensors located in CVOs, characterized by a more permeable BBB, are well positioned to respond rapidly.

Finally, it is important to consider and experimentally address why apparently redundant groups of nutrient-sensing neurons are situated at multiple loci across the neuroaxis. For example, leucine-sensing neurons have been identified both in the cmNTS and in the MBH (53; 54). Recapitulating sensing of the same nutrient at more than one site allows for additional temporal control: for example, to combine the predictive value of pre-absorptive nutrient sensing events supporting anticipatory responses with the direct detection of circulating nutrients by the brain in the post-ingestive period. Differentially distributed nutrient sensors might also respond preferentially during acute and rapid changes in nutrient levels, or instead following slow but sustained changes, as proposed in the context of brain sensing of hypoglycaemia (8).

## 2 How Does the Brain Sense Nutrients in a Unifying Manner?

As our understanding of the molecular, neuroanatomical, and neurochemical diversity of nutrient-sensing circuits continues to expand, the challenge becomes to understand how the brain integrates signals from distributed sensing sites to form a unified representation of nutrient status and to generate coherent behavioural and physiological feedback controls to restore nutrient homeostasis.

### 2.1 The Integrative nature of brain nutrient sensing

#### 2.1.1 Molecular integration of nutrient sensing within the neuron

Cell autonomous integration of nutrient sensing within a neuron relies on the ability of a neuron to directly detect, process and electrically respond to distinct meal-related cues. This should be distinguished from the neuromodulatory influence of cellular nutrient and energy availability, which might change the consequences of direct nutrient-sensing events for neuronal electrical activity. Such a distinction is not trivial, because direct neuronal nutrient sensing at least in part relies on the production of energy-related metabolites. Nevertheless, energy-dependant neuromodulation represents a distinct form of integration within the neuron.

Various classes of nutrients signal through shared intracellular pathways to regulate electrical activity in nutrient-sensing neurons. For example, glucose, oleic acid, leptin and insulin all modulate neuronal electrical activity through K_ATP_ channels (55–58). This suggests that K_ATP_-expressing neurons might integrate the detection of several classes of nutrients and hormones, but direct experimental evidence for this is limited. In fact, glucose and oleic acid mostly produce electrical responses in distinct populations of neurons in the ARH (59), but glucose availability exerts a neuromodulatory effect on the amplitude of neuronal electrical responses to oleic acid (59). In contrast, leptin and insulin regulate K_ATP_ channel activity independently of cellular energy levels, via membrane phospholipids (58). Insulin opens K_ATP_ channels in VMH glucose-sensing neurons, and reduces their neurophysiological response to hypoglycaemia (60), supporting the presence of neurons sensing both glucose and insulin.

Neuronal AMPK is a cellular energy sensor directly activated by decreased ATP concentration (61). In VMH glucose-sensing neurons, hypoglycaemia-induced activation of AMPK leads to nitric oxide (NO) production, closure of chloride channels and cell depolarisation (62). The orexigenic hormone ghrelin also signals via AMPK (63), proposed to integrate glucose and ghrelin sensing following the observation that the influence of ambient glucose concentration on ghrelin’s orexigenic action is abolished after knockdown of AMPK in AGRP neurons (64). However, just because glucose concentration modulates electrical responses to ghrelin does not mean that glucose and ghrelin sensing occur in the same neurons. Instead, AMPK activity might modulate the electrical and functional consequences of ghrelin signalling based on energy availability. New evidence indicates that AMPK is able to sense glucose availability independently of changes in adenine nucleotides (65). Such mechanistic insights will help establish the ability of nutrient-sensing neurons to neurophysiologically respond to multiple classes of nutrients. It is likely that low levels of other nutrients, such as amino acids, might also exert ubiquitous neuromodulatory effects on neuronal nutrient sensing, as suggested by the reduced functional consequences of leptin in mice lacking the neutral amino acid transporter LAT1 in neurons expressing the leptin receptor, mimicking amino acid deficiency (66).

Cellular sensing mechanisms upstream and independent from cellular energy metabolism contribute to fatty acid sensing through fatty-acid binding G protein coupled receptors (GPCRs) (67), L-leucine sensing via Cav3.1 (68), or glucose sensing through GPCRs (69). Future identification of neuronal nutrient-sensing mechanisms independent of cellular energetic pathways will facilitate progress in our understanding of how nutrient-sensing neurons might integrate distinct nutrient sensing events.

#### 2.1.2 Spatial integration of brain nutrient sensing at the circuit level

Neuroanatomical convergence of nutrient-sensing circuits creates opportunities for the integration of spatially-distributed nutrient-sensing events. One form of integration can occur through the innervation of nutrient-sensing neurons by upstream nutrient-sensing afferents. A classic example is the integration of viscerosensory vagal inputs within the cmNTS with nutrient-related signals sensed locally, modulating local vagal signal processing. Such integration is recruited for example to limit the appetite-suppressing effect of CCK in fasted rodents (70), or enhance CCK-induced satiation in response to increased L-leucine availability in the hindbrain (53). This type of integration modulates the amplitude of the responses to meal-related signals across various nutritional states, for example in mesolimbic dopaminergic circuits where food valuation is enhanced in fasted rodents through inputs of AGRP neurons to the VTA (71).

AGRP and POMC neurons, initially thought to function primarily as direct nutrient sensors, also receive sensory afferents, for example from food sensory detection (72) or hindbrain nutrient-sensing inputs. Inhibitory afferents to AGRP neurons are recruited in response to nutrient sensing in the cmNTS, delaying dark-onset meal initiation (73), while excitatory afferents from cmNTS glucose-inhibited neurons are recruited for glucoprivic feeding (74). Integration of glucose-sensing afferents by glucose-sensing neurons has been described in other circuits, for example VMH glucose-sensing neurons receiving inputs from glucose-inhibited lateral parabrachial nucleus (PBN) neurons during hypoglycaemia to control neuroendocrine counterregulatory responses (75). Results from studies of chemogenetic inhibition of various arms of this circuit suggests that hypoglycemia sensing in both sites is required for a full neuroendocrine response to glucoprivation (75). (need citation here?).

Spatial integration can also occur in neuronal populations receiving multiple converging afferents from nutrient-sensing neurons. The PVN, PBN and cmNTS, for example, receive inputs from distributed nutrient-sensing neurons (76–78). PVH neuronal populations exhibit marked heterogeneity (79) and whether individual PVH neurons integrate information from distributed nutrient-sensing afferents remains unclear. In the PBN, aversive, non-aversive or glucoregulatory inputs appear to be segregated (80). Likewise, cmNTS neurons respond to distinct mechanical or chemical inputs (50; 81), and recruit distinct downstream circuits in response to gut glucose- or fat-sensing inputs ((46; 82). Thus, although nutrient sensing circuits converge at common brain nuclei, advanced neuroanatomical, molecular and functional investigations suggest the existence of distinct nutrient-sensing circuits for various sensory modalities within these nuclei (81). Such organisation might facilitate the production of super-additive responses, as seen in response to gut sugar and lipid sensing (82). New weight-loss therapies, including trizepatide or retatrutide may leverage such superadditive appetite-inhibiting responses produced by combinations of molecules targeting distinct nutrient-sensing pathways, highlighting the potential clinical impact of further studies characterising the mechanisms underpinning circuit-level integration of meal-related inputs.

#### 2.1.3 Temporal integration of brain nutrient sensing

The increasing use of in vivo recording technologies to track the activity of molecularly defined neuronal populations before, during, and after food ingestion is shedding light on the temporal regulation of nutrient-sensing pathways. For example, AGRP neurons are rapidly inhibited by pre-ingestive detection of food sensory cues. Feeding transiently restores their activity to baseline, and post-ingestive gut nutrient detection is necessary to sustain AGRP suppression in response to food consumption (83). However, long-term energy balance requires persistent suppression of AGRP activity through direct nutrient sensing (50; 83).

#### 2.1.4 Integration of nutrient-sensing in memory circuits

Meal-related signals influence memory, which in turn contributes to the control of feeding behaviour (84). The hippocampus, a critical neurobiological substrate of memory processing, is activated by meal-related signals (85) and required for hunger suppression following a meal (86). Hippocampal detection of meal-related signals such as ghrelin or nutrient-sensitive afferent input also increases the motivation to eat (87), suggesting a role for hippocampal circuits in both homeostatic and hedonic dimensions of feeding behaviours. In this regard, hippocampal to lateral septum signalling has been proposed to encode appetitive spatial memory of past feeding events (88). In addition, memory consolidation during sleep has been proposed to stabilise the hypothalamic representation of feeding behavior and modulate future food intake. Specifically, during REM sleep, the sleep phase essential for learning and memory consolidation, LH neurons recruited during feeding are reactivated, and this reactivation promotes subsequent feeding behaviours (89; 90).

### 2.2 Coordination of central and peripheral effectors by brain nutrient-sensing circuits

In response to meal-related signals, the brain engages various feedback responses that determine nutrient uptake, storage, synthesis, and breakdown across distributed peripheral tissues and throughout the feeding sequence. Precise regulation of the onset, duration, and amplitude of these responses is essential for the rapid restoration and maintenance of nutritional balance.

A common view is that brain nutrient-sensing circuits coordinate multiple effector output pathways. Is this coordination an emergent property of independent, parallel pathways recruited by meal-related signals, or have mammalian brains evolved specialized mechanisms to integrate and synchronize these outputs? Neuroanatomical studies using retrograde polysynaptic tracing viruses injected into different peripheral organs suggest the predominance of the latter. Results from these investigations reveal neuronal populations wired for the simultaneous regulation of multiple output circuits and peripheral targets. For instance, subsets of ARH, LH, PVH, cmNTS, and dorsal motor nucleus of the vagus (DMV) neurons send descending projections to both the liver and pancreas, or to both the liver and intra-abdominal fat (91; 92). Similarly, neurons from the insular cortex, amygdala, lateral hypothalamus, and ectorhinal/perirhinal cortices send collaterals to the masseter muscle, salivary glands, and tongue (93). Moreover, subsets of RVM neurons innervate both sympathetic and motor-related spinal cord regions, suggesting a potential for coordinated somatic and autonomic control (94). Central circuits that would allow the coordination of nutrient-sensing output circuits remain poorly documented. The DMV and PVH are both well positioned to potentially coordinate sympathetic, parasympathetic, neuroendocrine or somatic motor neurons (95; 96). However, evidence regarding the presence of cmNTS neurons projecting collaterals to distinct forebrain sites is conflicting. Some studies report that subsets of PPG and catecholaminergic neurons send collaterals to the PVH and BST (97; 98). In contrast, other findings suggest that most cmNTS neurons project unilaterally to single autonomic targets, lacking collateralization to the PVN, PBN, and caudal VLM (99). As for the PVH, one tracing study indicates that 30% of PVN neurons projecting to the IML also innervate sympathetic premotor neurons in the rostral ventrolateral medulla (100). Thus, further research is essential to determine the functional relevance of these projections in regulating peripheral effectors, and to assess the degree to which nutrient-related signals engage these neuronal populations to restore nutrient homeostasis.

In contrast to the central projection circuits described above, a comprehensive neuroanatomical and functional characterization of the output of the peripheral superior mesenteric ganglia reveals selective and mutually exclusive axonal projections to the gastrointestinal tract, pancreas, or bile ducts, supporting the functional segregation of sympathetic motor neurons (101). Similar segregation is observed in hypothalamic nutrient-sensing circuits. For example, segregated subsets of LH^VGAT^ neurons exhibit distinct activity patterns during food intake and food approach (89). Segregated populations of AGRP and POMC neurons send unique projections to either the bed nucleus of the stria terminalis, PVH, LH, paraventricular nucleus of the thalamus, central nucleus of the amygdala, or periaqueductal gray (102). This suggests that different branches of the melanocortinergic system specialize in regulating structurally and functionally distinct downstream circuits. Functional segregation within melanocortinergic pathways is further supported by results from studies in melanocortin receptor 4 (*Mc4r*) knockout mice, where selective *Mc4r* reintroduction into parasympathetic versus sympathetic neurons produces distinct physiological effects on energy expenditure vs. hepatic glucose output (103).

While growing evidence supports the segregation of nutrient-sensing output circuits, it remains unclear how brain nutrient-sensing circuits coordinate the regulation of anatomically distinct peripheral and central targets. One proposed mechanism is neuropeptide volume transmission, either locally or through the cerebrospinal fluid, enabling simultaneous activation of multiple targets in a one-to-many mode (104). This mechanism has been implicated in the recruitment of molecularly diverse neuronal populations across behavioral states (79). For instance, single-cell transcriptomic analyses combined with in vivo calcium imaging identified NPY1R expression as a key predictor of PVH neuronal ensemble activation across diverse molecular subtypes (79). Given the prevalence of volume transmission for monoamines and neuropeptides, this mechanism may be particularly relevant in the hypothalamus, where many neuronal populations primarily use neuropeptides as transmitters (105). Hypothalamic neurons projecting to ependymal cells could leverage volume transmission to activate distal targets over extended periods, as proposed for PVH oxytocin neurons (106) or LH MCH neurons (107). Volume transmission also facilitates sustained long-range communication between hypothalamic neuroendocrine circuits and hindbrain structures, for example, mediating long-lasting cortical excitability following acute stress (108).

Another possible coordination mechanism involves circuits connecting various branches of the autonomic nervous system. For example, local activation of inguinal adipose tissue afferents triggers brown fat thermogenesis (109). This sensory-sympathetic motor crosstalk is mediated by groups of neurons distributed across the hypothalamus, hindbrain, the spinal cord and sympathetic and dorsal ganglia, receiving afferent inputs from white adipose pads and projections to the brown fat projecting (or vice-versa) (110; 111). In addition, interneurons bridging parasympathetic and sympathetic efferent pathways have been identified, with pancreas-injected polysynaptic anterograde tracers appearing in the IML following sympathectomy or in the DMC following vagotomy (112).

Finally, synchronized oscillatory activity may mediate the coordinated control of distributed output circuits in response to nutrient sensing. Gamma oscillation is proposed to underlie computation across brain circuits, modulating action potential timing, neuronal population synchrony, and cross-structural communications (113). Emerging evidence is supporting a role for gamma oscillations in the control of feeding behaviour. The prefrontal cortex, septum, and lateral hypothalamus synchronize at gamma rhythms to regulate feeding-related behaviors (114), and LH neurons fire together with greater probability during slow and fast gamma oscillations compared to non-rhythmic periods (90). Synchronised activity in feeding circuits might increase the robustness of feeding behaviour, as seen during cocaine-paired memory acquisition (115) or in response to food-context hyperphagia (116). Further work is needed to identify putative roles for brain oscillations in the coordination of effector output circuits recruited in response to brain nutrient sensing.

## 3 Why? The Physiological Relevance of Brain Nutrient Sensing: Metabolic Health and Beyond

In this last section, we examine evidence supporting roles for brain nutrient sensing in the maintenance of nutrient homeostasis during nutrient deficit or surfeit, and its relevance as a therapeutic target for metabolic and neurodegenerative diseases.

### 3.1 Brain nutrient sensing to face periods of nutrient deficit

The essential role of the brain in producing homeostatic feedback responses to energy deficit is well established and believed to work largely through the pleiotropic consequences of decreased central leptin signalling (117). Is brain nutrient sensing also engaging homeostatic feedback loops to maintain circulating availability of essential nutrients? As highlighted with the examples discussed so far, there is strong evidence for a role of brain glucose sensing in the systemic response to glucose deficit (118). cmNTS catecholaminergic glucose-sensing neurons have been proposed to mediate glucoprivic feeding through their projections to the ARH (74), while glucose-sensing neurons in the VMH are crucial in the autonomic and neuroendocrine controls of blood glucose during hypoglycaemia. VMH-specific glucoprivation or optogenetic activation of VMH glucose-sensing neurons produces hyperglycaemia through robust increases in circulating levels of counterregulatory hormones (119). Conversely, glucose infusion in the VMH or photoinhibition of VMH glucose sensing neurons during a hypoglycemic clamp blocks autonomic and neuroendocrine responses (120; 121).

These studies use extreme hypoglycaemic challenges not likely to be encountered physiologically. They identify the capabilities but not the natural functions of glucose-sensing networks. The extent to which these circuits are recruited in normal physiology warrants additional investigation, for example, using in vivo neuronal recordings during physiological hypoglycaemia. This strategy has been used to link changes in blood glucose levels to changes in the activity of VMH PACAP neurons, whose activity negatively correlates with blood glucose levels (122). The role of decreased glycaemia in meal initiation is one of the premises of the glucostatic theory of feeding (2), finding some recent support in humans with the observation that a dip in glycemia after a meal is a significant predictor of hunger and future caloric intake (123). However, microdialysis measures of parenchymal glucose in the VMH failed to detect drops in VMH glucose concentrations before meal onset (3). In addition, the relative contribution of neuroendocrine and feeding circuits in response to hypoglycaemia should be considered, bearing in mind that the hormonal responses are observed more rapidly and reproducibly than the feeding response, and at lower threshold doses of 2-deoxyglucose (124). Thus, we still do not fully understand how brain glucose sensing contributes to responses to decreased peripheral glucose availability under physiological conditions.

Evidence supporting a direct role for brain fatty acid sensing in peripheral lipid handling during fasting or specific fatty acid deficiency is limited, despite the established importance of neuroendocrine output and the autonomic nervous system in the control of lipid metabolism in the liver, and white and brown adipose tissue (125). Brain detection of systemic energy deficit can recruit peripheral lipid handling pathways, as indicated by the lipolytic effect of peripheral or central leptin injection (126) and the increased lipolysis and decreased de novo lipogenesis in the white adipose tissue of mice lack the insulin receptor in neurons (127). Likewise, the activation of the HPA axis during energy deficit has profound consequences on peripheral lipid metabolism (128). However, the recruitment of these pathways following activation of brain fatty acid sensing circuits is unclear. Genetic deletion of the fatty sensing receptor CD36 in the hypothalamus blocks the anorectic response to intracarotid fatty acid delivery in rats (129), but the effects on peripheral lipid handling have not been identified.

In contrast, strong evidence implicates brain sensing of protein deficit in the production of protein-specific appetite and protein preference, and these effects are believed to be mediated by central FGF21 signalling (130). FGF21 also promotes muscle atrophy and increased circulating levels of amino acids (131), but the contribution of brain protein sensing to these responses is unclear. Intriguingly, mice exposed once to protein restriction maintain long term preferences for protein-rich diets, suggesting that protein sensing promotes long-term adaptive changes in brain feeding circuits to promote amino acid homeostasis (130).

### 3.2 Brain nutrient sensing to optimise internal nutrient utilization and future nutrient acquisition

A return to nutrient homeostasis after a meal can be viewed as strategy to primarily maintain stable nutrient availability over a longer inter-meal interval, allowing efficient and sparing management of internal nutritional resources in the face of a nutritionally variable or insecure external environment. There is strong support for the idea that brain glucose sensing during postprandial hyperglycaemia promotes a return to euglycaemia through the suppression of food intake (132), hepatic glucose production (133), and the activation of insulin secretion (134). We will not expand on this aspect of the literature that is less contentious and has been reviewed before (51).

However, a number of studies using lipid or fatty acid injections or manipulating brain lipid sensing pathways support the idea that brain lipid sensing also produces feedback responses allowing the maintenance of peripheral lipid homeostasis. Intracarotid triglyceride infusion rapidly reduces food intake through hypothalamic CD36 (129), and this anorectic response is also observed in response to intra-cerebroventricular or hypothalamic oleate injection (135; 136). Reduced food intake in response to brain lipid excess could reflect a strategy to avoid further ingestion of lipid, or more specifically the production of lipid avoidance. This is suggested by the inhibition of food reward following central activation of FFAR4 (137). In contrast, VTA FFAR4 activation does not modulate place preference (67), leaving unclear the central mechanisms mediating this effect. Hypothalamic fatty acid injections also promote insulin secretion (138), the synthesis of hepatic very low-density lipoproteins (139), and the sympathetic control of brown fat thermogenesis, a triglyceride sink, and white adipose tissue beiging (140–142). Manipulation of brain lipogenesis through deletion of neuronal lipoprotein lipase mimics some of these responses (141), but this produces widespread consequences on brain lipid availability in general, beyond interfering with the activity of any specific lipid-sensing neuronal populations (141). Nevertheless, inhibition of hypothalamic lipogenesis blunts thyroid hormone induced activation of brown fat thermogenesis (143), highlighting an interaction with neuroendocrine pathways. Collectively, these responses contribute to a return to baseline lipid homeostasis, but more research is needed to bridge the gap between molecular lipid-sensing events and the activation of autonomic and neuroendocrine pathways that regulate peripheral lipid metabolism.

Our current understanding of the physiological roles of brain amino acid sensing during amino acid excess is largely limited to its function in appetite regulation. This has been demonstrated through brain injections of the amino acid leucine or manipulation of the amino acid-sensing pathway mTOR (mammalian target of rapamycin) in the hypothalamus and brainstem (53; 54). In addition, hypothalamic amino acid sensing increases sympathetic tone to brown fat and brown fat thermogenesis (144), which could serve as a sink for circulating amino acids in conditions of excess (145). Hypothalamic leucine sensing increases hepatic gluconeogenesis (146), another mechanism of amino acid disposal, but the consequences on circulating amino acid availability have not been examined. Future research should focus on the identification of potential outcomes of lecuine sensing on peripheral amino acid metabolism. Notably, glucagon is an important signal for the regulation of systemic amino acid availability (147) regulated by parasympathetic output (148), but whether brain amino acid sensing modulates pancreatic glucagon release is unknown.

Despite these gaps, there is overall some support for the idea that brain sensing of an excess in a specific nutrient triggers negative feedback responses to suppress the intake of and increase the utilisation/storage of that specific nutrient. Of note, brain sensing of fatty acid or amino acid excess also modifies peripheral glucose handling (146; 149), suggesting that sensing of an individual nutrient influences peripheral handling of other nutrients, promoting overall nutritional homeostasis. This might reflect the fact that nutrients are rarely in excess on their own in naturalistic conditions, but instead reflect the ingestion of mixed meals. Accordingly, it is important to experimentally extend this concept to examine whether brain leucine sensing modulates peripheral utilization of other amino acids as well.

In contrast to these data supporting a role for brain nutrient sensing in generating negative feedback responses promoting a return to homeostasis, the central nervous system processing of peripheral lipid sensing inputs can activate hedonic circuits and further promote lipid intake. For example, activation of FFAR4 signaling in lingual taste buds increases 24-hour fat preference (150). Gut and brain lipid sensing also recruit VTA dopaminergic signaling, promoting fat preference and intake (82; 151). How anorectic and hedonic processing of brain lipid sensing are integrated is unclear. One possibility would be that initial lipid sensing in the periphery during ingestion promotes intake, while the appetite-suppressing consequences of brain lipid sensing emerge in the post-ingestive period, once brain fatty acid concentrations exceed physiological levels. Interestingly, recent evidence demonstrates that sugar and fat sensing by the gut-brain axis, processed through mesolimbic pathways, activates memory circuits for the formation of contextual memories associated with the consumption of energy-dense foods (87). This suggests brain nutrient sensing also promotes cognitive processes to optimize responses to future nutrient shortage, serving long-term nutrient homeostasis.

### 3.3 Brain nutrient sensing: beyond nutrient homeostasis

Brain glucose sensing is compromised in obesity and type 2 diabetes, due at least in part to blunted brain glucose access and uptake during hyperglycemia (152) and reduced neuronal glucose-sensing activity (153). Conversely, impairments in brain glucose sensing, such as through the deletion of neuronal *Glut2* or *Gck*, adversely affect peripheral glucose regulation (154; 155), suggesting a causal link between reduced brain glucose sensing and the development of dysfunctional glycemic control. Notably, studies have shown that selective activation of glucose-sensing neurons, which typically respond to low glucose levels, can induce hyperglycemia independently of peripheral insulin action (75). This provides a mechanistic framework supporting the role of impaired brain glucose sensing in the pathogenesis of diabetes. Remarkably, central pharmacological interventions, such as hypothalamic FGF1 injections, have been shown to reverse diabetes in genetically diabetic rodents or those rendered diabetic through streptozotocin treatment (156). These findings further underscore the significance of brain glucose-sensing circuits in determining blood glucose levels and highlight their potential as targets for antidiabetic therapies.

The consequences of obesity or chronic hypertriglyceridemia on the activity of fatty acid sensing neurons are not well characterised. One study found decreased excitability of POMC fatty-acid sensing neurons in high-fat fed mice, but no change in neuronal responses to oleic acid (56). However, brain uptake of radiolabelled fatty acid is doubled in obese patients (157), suggesting ectopic fat accumulation in the brain, a condition that promotes the development of lipotoxicity and obesity-associated complications (158). In fact, hypothalamic synthesis of palmitate and its product ceramide increase in obese rodents and promote insulin resistance and weight gain (159; 160). Moreover, blockade of ceramide synthesis restores metabolic health in obese rodents (161) but our understanding of how this functional rescue occurs remains limited. While these data support a role for impaired brain lipid metabolism in obesity, further work in needed to determine whether impaired brain lipid sensing could shift the response thresholds for neuronal responses to lipids, leading to impaired peripheral lipid handling and increased circulating lipid levels, akin to an increase in the defended level of blood lipids.

Likewise, little is known about how brain protein sensing might adapt in conditions of protein excess, and whether impaired brain protein sensing might produce impairments in peripheral protein metabolism. Muscle wasting in the absence of malnutrition occurs in diseases such as cancer (162), or during weight loss (163), but little is known about how brain protein sensing circuits might contribute to the increased proteolytic activity in skeletal muscle under these conditions. Cancer cachexia is also associated with reduced hunger, mostly attributable to increased inflammation in brain appetite-regulating circuits in response to tumour-released pro-inflammatory signals (164), but no direct link to brain protein sensing has been established. Brain-body interactions are clearly impaired in this pathological state, with increased stress axis activity and elevated sympathetic tone to brown and white fat, leading to lipolysis, WAT browning, and increased metabolic rate (165). Endocrine mediators such as GDF15 have been identified and proposed as efficient therapeutic targets in cancer cachexia, but how they might link to protein homeostasis is unclear (166).

Impaired brain nutrient sensing during overnutrition has widespread effects on the brain, with neurons being particularly vulnerable to pro-oxidative and inflammatory damage. Increasing evidence suggests that impaired nutrient sensing contributes to neurodegenerative diseases, with obesity recognized as a significant risk factor for multiple forms of dementia and multiple sclerosis (167). This association may reflect the ubiquitous and cell-autonomous effects of nutrient excess on cellular dysfunction, disrupting neurogenic, gliogenic, and repair processes—common features of neurodegenerative states (168). However, neurodegenerative diseases are now understood as complex pathological processes that begin decades before cognitive decline becomes apparent. Emerging evidence suggests that impaired brain nutrient-sensing pathways may play a role in the etiology of these diseases. For instance, early cellular defects in hypothalamic structures, such as the PVH and the suprachiasmatic nucleus (SCN), have been reported in the brains of Alzheimer’s disease patients, independent of amyloid-beta peptide deposition (169). These hypothalamic structures exhibit early and pronounced vulnerability in Alzheimer’s disease (170). Such early alterations have been proposed to mediate impairments in neuroendocrine and autonomic functions at the initial stages of the disease (171). In Alzheimer’s disease, metabolic abnormalities—such as alterations in body weight and neuroendocrine function—often precede cognitive decline and are considered integral to the pathophysiological process (172). Similarly, in multiple sclerosis, hypothalamic lesions are common (173), and impaired hypothalamic activity occurs early in disease progression, notably disrupting the hypothalamic-pituitary-adrenal (HPA) axis (174).

Longitudinal studies in large human cohorts, such as the UK Biobank, have reported that an earlier age at hyperlipidemia diagnosis is significantly associated with a higher risk of all-cause dementia (175), This finding suggests that impaired lipid homeostasis may contribute to neurodegenerative processes. Additionally, hypercholesterolemia increases the future risk of dementia, and lipid-lowering treatments have been shown to improve dementia symptoms (176). In multiple sclerosis, elevated lipoprotein lipase (LPL) autoantibodies and increased circulating triglyceride levels have been observed, and lipid-lowering therapies also appear to improve symptoms (177). Likewise, several forms of dementia are associated with impaired amino acid homeostasis (178). In the UK Biobank, branched-chain amino acids (BCAAs) have been linked to an increased risk of dementia, Alzheimer’s disease, and Parkinson’s disease (179). While this association could be a consequence of disease progression (180), future studies should investigate the role of impaired brain nutrient sensing in the early stages of neurodegeneration. Exploring the connections among brain nutrient sensing, peripheral nutrient homeostasis, and neurodegenerative disease could provide novel insights into disease mechanisms and potential therapeutic targets.

In fact, lifestyle and pharmacological strategies primarily affecting nutrient sensing pathways, such as caloric restriction, metformin or GLP1 receptor agonists, are being evaluated for the treatment of various neurodegenerative diseases. Caloric restriction and fasting mimetics such as metformin counteract the progression of neurodegenerative diseases and promote brain repair in animal models of Alzheimer’s and Parkinson’s diseases or multiple sclerosis (181; 182). Randomised controlled trials in patients with multiple sclerosis suggest significant cognitive improvements with caloric restriction (183) and ongoing clinical trials are evaluating the effect of metformin treatment on cognitive functions in patients with neurodegenerartion (184). Likewise, GLP1 agonists exhibit beneficial effects on neurodegeneration, motor function and cognition in animal models of Alzheimer’s and Parkinson’s diseases (185). In the healthy ageing populations and people with dementias, GLP1R agonists improve memory functions (186) and are being evaluated for the treatment of Alzheimer’s disease (187). Strikingly, GLP1R agonists such as semaglutide or trizepatide do not cross the blood-brain barrier, and only signal in brain areas expressing fenestrated capillaries, the CVOs, highly enriched in nutrient sensing cells (188). This critical observation raises the possibility that improved nutrient homeostasis might be a primary mechanism responsible for their beneficial anti-neurodegenerative effects. In fact, in addition to an increased occurrence of neurodegenerative diseases, aging also associates with impaired homeostatic control of blood glucose, amino acids and lipids (189–191). Taken together, these findings support the suggestion that impaired nutrient homeostasis contributes to the development of neurodegenerative disorders, and underscore the importance of future work targeting nutrient sensing deficiency in neurodegeneration as a therapeutic approach.

### Concluding remarks

1) More work is needed to identify how non-neuronal cells contribute to brain nutrient sensing independently of their role in neuronal energetic support and energy-dependent neuromodulation.
2) The roles for brain nutrient sensing in fatty acid and amino acid systemic homeostasis require further empirical support - future work can apply the current conceptual framework to design more incisive and revealing experiments, offering new ways of interrogating the physiological roles of brain nutrient sensing.
3) The contribution of impaired brain nutrient sensing to the pathogenesis of diseases associated with dysfunctional peripheral nutrient homeostasis, such as obesity, type 2 diabetes, hyperlipidemia, or muscle wasting in cachexia and malnutrition, should be evaluated. Further experimental interrogations of brain nutrient sensing pathways in disease states might uncover new therapeutic strategies for conditions associated with dysregulated nutrient homeostasis.
4) It remains critical to identify the extent to which cognitive effectors are important targets for return to homeostasis – to restore feedback that the system requires for effective short- and long-term regulation of nutrient availability.

## Figures and Tables

**Figure 2:**
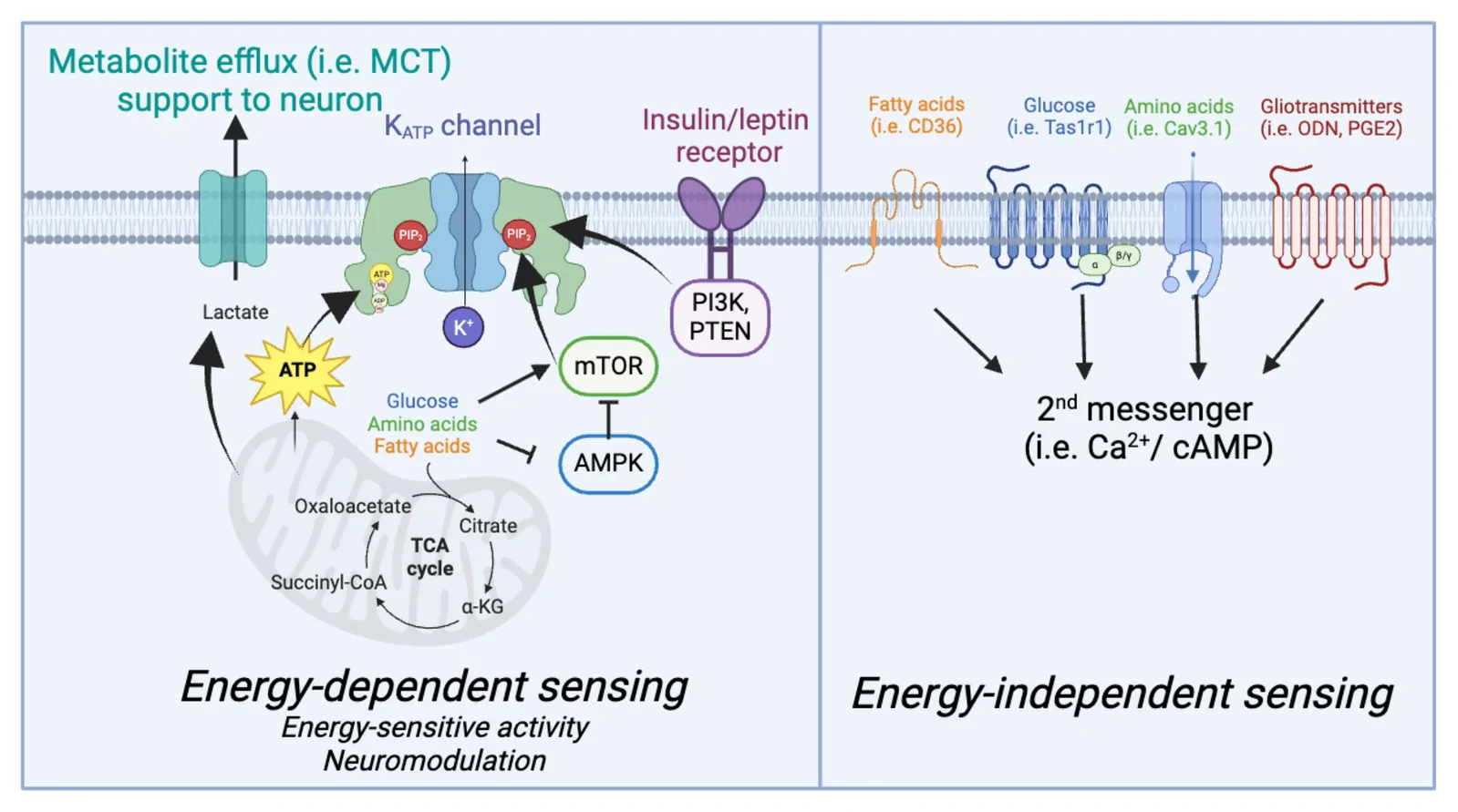
Brain nutrient detection: Sensing versus fuelling. Nutrients recruit energy-dependent intracellular mechanisms to change the functional output of nutrient-sensing cells. Energy-dependant sensing mechanisms also provide neuromodulatory inputs, changing the consequences of other nutrient-sensing events in the nutrient-sensing niche. Energy-independent sensing pathways alter the functional output of nutrient-sensing cells independently of fuel availability. MCT: monocarboxylate transporter

**Figure 3:**
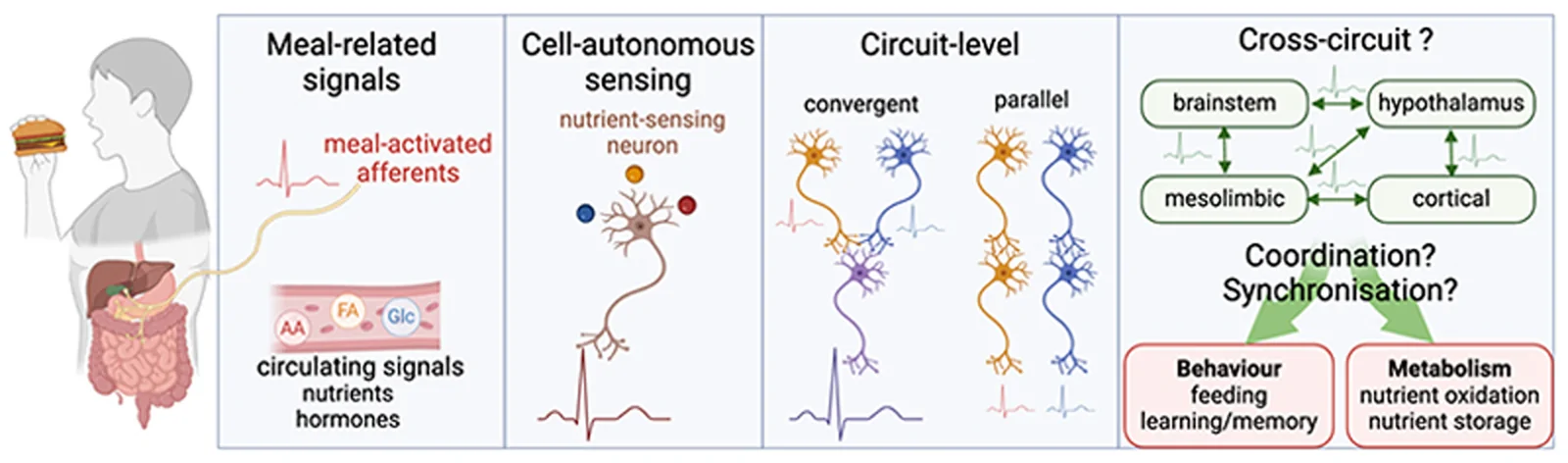
Brain integration of nutrient-sensing inputs. Circulating meal-related signals and ascending nutrient-sensing afferents alter the neurophysiolgical activity of distributed populations of brain nutrient-sensing neurons, which regulate downstream neural circuits for behavioural and metabolic feedback controls. Detection of different meal-related inputs can be integrated at the cell autonomous level within nutrient-sensing neurons sensitive to different sensing modalities, or at the circuit level in neuronal populations receiving convergent nutrient-sensing inputs. Future study is critical to identify and characterize parallel and distinct nutrient-sensing inputs, and to understand how activity across circuits may be integrated to generate a unified control of behavioural and metabolic circuits for short- and long-term nutrient homeostasis.

## Data Availability

This article has no additional data.
